# Identification of hub methylated‐CpG sites and associated genes in oral squamous cell carcinoma

**DOI:** 10.1002/cam4.2969

**Published:** 2020-03-10

**Authors:** Yuxin Dai, Qiaoli Lv, Tingting Qi, Jian Qu, Hongli Ni, Yongkang Liao, Peng Liu, Qiang Qu

**Affiliations:** ^1^ Department of Pharmacy Xiangya Hospital Central South University Changsha Hunan China; ^2^ Department of Biochemistry and Molecular Biology School of Life Sciences Central South University Changsha Hunan China; ^3^ Department of Science and Education Jiangxi Key Laboratory of Translational Cancer Research Jiangxi Cancer Hospital Nanchang Jiangxi China; ^4^ Department of Pharmacy The Second Xiangya Hospital Central South University Changsha Hunan China; ^5^ College of Bioscience and Biotechnology Hunan Agricultural University Changsha Hunan China; ^6^ Institute for Rational and Safe Medication Practices National Clinical Research Center for Geriatric Disorders Xiangya Hospital Central South University Changsha Hunan China

**Keywords:** CpG site, methylation, multiomics, oral squamous cell carcinoma, survival analysis, WGCNA

## Abstract

To improve personalized diagnosis and prognosis for oral squamous cell carcinoma (OSCC) by identification of hub methylated‐CpG sites and associated genes, weighted gene comethylation network analysis (WGCNA) was performed to examine and identify hub modules and CpG sites correlated with OSCC. Here, WGCNA modeling yielded blue and brown comethylation modules that were significantly associated with OSCC status. Following screening of the differentially expressed genes (DEGs) from gene expression microarrays and differentially methylated‐CpG sites (DCGs), integrated multiomics analysis of the DEGs, DCGs, and hub CpG sites from the modules was performed to investigate their correlations. Expression levels of 16 CpG sites‐associated genes were negatively correlated with methylation patterns of promoter. Moreover, Kaplan‐Meier survival analysis of the hub CpG sites and associated genes was carried out using 2 public databases, MethSurv and GEPIA. Only 5 genes, *ACTA1*, *ACTN2*, *OSR1*, *SYNGR1*, and *ZNF677*, had significant overall survival using GEPIA. Hypermethylated‐CpG sites ACTN2‐cg21376883 and OSR1‐cg06509239 were found to be associated with poor survival by MethSurv. Methylation status of specific site and expression levels of associated genes were determined using clinical samples by quantitative methylation‐specific PCR and real‐time PCR. Pearson's correlation analysis showed that methylation levels of cg06509239 and cg18335068 were negatively related to *OSR1* and *ZNF677* expression levels, respectively. Our classification schema using multiomics analysis represents a screening framework for identification of hub CpG sites and associated genes.

## Graphical abstract

Construction of comethylation network associated with oral squamous cell carcinoma status by WGCNA. Dendrogram of consensus module eigengenes obtained by WGCNA on the consensus correlation. Seven modules were generated after merging according to the correlation of modules.

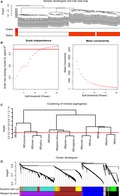

## 1 INTRODUCTION

Oral cancers represent the sixth‐most common malignant tumor type.^1^ Oral squamous cell carcinoma (OSCC) accounts for 90% of the incidence of head and neck squamous cell carcinoma (HNSCC) and has a poor prognosis and low 5‐year survival rate.^2^ Smoking, drinking, and chewing betel nuts are the main causes of OSCC; however, its tumorigenesis is a complicated and multistep process involving many crucial genes and signaling pathways.^3^, ^4^, ^5^ It is, therefore, necessary to explore the underlying molecular biology mechanisms of the pathogenesis of OSCC and further characterize its early prognostic signature in order to identify novel drug targets for the disease.^6^, ^7^

Over the past few decades, early epigenetic changes have been reported to predispose cells to further genetic abnormalities, which may allow progression of the neoplastic process.^8^, ^9^ Epigenetic modification, particularly DNA methylation regulation, in carcinogenesis has become a focus of cancer research.^10^, ^11^ Numerous studies have shown that aberrant DNA methylation can lead to changes in chromatin structure, DNA conformation, DNA stability, and DNA‐protein interactions, thereby dysregulating gene expression.^12^ Aberrant methylation patterns of CpG sites within gene promoters have been shown to be correlated with the development of human cancers, including OSCC.^8^, ^12^, ^13^, ^14^ These findings indicate that the differential methylation phenotype of CpG islands may be an early event in OSCC development.^15^ *AGTR1*, *FOXI2*, and *PENK* promoter hypermethylation and *LINE1* hypomethylation may be associated with an increased risk of OSCC development.^15^ In addition, the methylation status of certain genes, including *FLT4*, *KDR,* and *TFPI2*, could potentially be used as a biomarker for early detection of OSCC.^8^ Thus, there is a motivation to further explore the correlation between the methylation status of CpG sites and associated gene expression in OSCC.

Weighted gene coexpression network analysis (WGCNA) is a typical systematic biological algorithm that aims to separate and identify core genes significantly related to certain biological traits from a dataset consisting of thousands of genes.^16^, ^17^ WGCNA has been used to identify hub genes, microRNAs (miRNAs), and long noncoding RNAs (lncRNAs) in a wide variety of cancers, including osteosarcoma,^18^ glioblastoma,^19^ colon cancer,^20^ breast cancer,^21^ ovarian cancer,^22^ and cholangiocarcinoma.^23^ Moreover, these hub genes, miRNAs and lncRNAs, were found to be significantly correlated with progression, prognosis, metastasis, and recurrence in multiple carcinomas. Recently, in order to determine the correlation between hub methylated‐CpG sites and clinical traits of cancers, a modified weighted gene comethylation network analysis (WGCNA) was performed on methylation microarrays of large samples.^10^, ^24^, ^25^ Based on the analysis of hub genes related to radiotherapy efficacy by WGCNA, a 4‐gene methylation signature was identified that could predict survival outcomes of patients with HNSCC.^26^ However, further evidence is required to determine whether methylated‐CpG sites and their corresponding genes affect the tumorigenesis and development of OSCC.

In this study, we aimed to identify hub differential methylated‐CpG sites and associated genes in OSCC through systematic multiomics analysis, using WGCNA, CpG methylation profiles, and mRNA expression profiles from the Gene Expression Omnibus (GEO) database and other public databases. We also investigated CpG sites and gene‐based prognostic signatures based on accurate prediction of overall survival by public database MethSurv and GEPIA (Gene Expression Profiling Interactive Analysis). Finally, we developed a modified quantitative methylation‐specific PCR (QMSP) to determine methylation status of specific CpG sites. We determined the correlation of hub CpG sites and mRNA levels of associated genes. Our findings indicate that integrated multiomics analysis is a favorable method for identifying hub genes and differential CpG sites, which can be expected to provide novel insights for screening prognostic and diagnostic indicators of OSCC in the future.

## 2 MATERIAL AND METHODS

### 2.1 Data collection

All microarray data were downloaded from the GEO database (http://www.ncbi.nlm.nih.gov/geo/). The methylation microarray (http://www.ncbi.nlm.nih.gov/geo/query/acc.cgi?acc=GSE38532) included 40 OSCC samples and 40 nontumor pairwise samples, based on the Illumina HumanMethylation27 BeadChip (GPL8490 platform). The gene expression microarray (http://www.ncbi.nlm.nih.gov/geo/query/acc.cgi?acc=GSE30784) used as the training dataset contained 167 OSCC samples and 45 normal oral tissues. The gene expression microarray (http://www.ncbi.nlm.nih.gov/geo/query/acc.cgi?acc=GSE31056) used for validation contained samples from 21 OSCC patients and 24 normal tissue samples. The Affymetrix Human Genome U133 Plus 2.0 array was used to annotate the probes in both cases. This integrated study was a second analysis of GEO data; as such, patient consent was not required.

### 2.2 Construction of weighted gene comethylation network

To illustrate our workflow of identification of hub methylated‐CpG sites and associated genes in OSCC, overall flowchart about analysis pipeline was in the Figure S1. The WGCNA algorithm was used to construct comethylation networks.^16^ The methylation microarray http://www.ncbi.nlm.nih.gov/geo/query/acc.cgi?acc=GSE38532 containing 80 samples comprised a matrix of methylation CpG sites. First, before network construction, obvious outlier samples with excessive numbers of missing entries were removed using a sample dendrogram cluster method for outlier detection, with visualization using a trait heatmap. Second, the soft‐threshold power was selected using the pickSoftThreshold function of WGCNA based on the criteria of approximate scale‐free topology and mean connectivity. Third, we calculated adjacencies using best‐fit soft‐threshold power, transformed the adjacency into a topological overlap matrix (TOM), and calculated the corresponding dissimilarity TOM (dissTOM). Finally, after hierarchical clustering analysis based on the dissTOM, modules were generated by the dynamic tree cut method for module merging (deep split = 2, cut height = 0.25; default values were used for the other parameters).

### 2.3 Construction of module‐trait relationships and identification of hub CpG sites associated with clinical modules

Pearson's correlation coefficients and *P*‐values between modules and OSCC status were calculated and visualized in a module‐trait heat map. Module membership (MM) represents the correlation of the methylation patterns of CpG sites with OSCC clinical status. The average gene significance (GS) in each module (module significance, MS, is the absolute average value of the correlation between all the CpGs and the trait) was displayed in a bar graph, which also reflected the relationship between the module and OSCC clinical status. Modules with the highest MS and MM were considered to be key modules with respect to OSCC status. To further identify hub CpG sites within key modules, a scatter plot of GS and MM for all CpG sites within each module was constructed. Using MM >0.8 and GS >0.8 as screening criteria, hub CpG sites were selected from among the key modules, and associated genes were annotated for subsequent function enrichment analysis.

### 2.4 Function enrichment analysis for hub CpG site‐associated genes from key modules

Enrichr (https://amp.pharm.mssm.edu/Enrichr/) is a web‐based tool for functional enrichment analysis of signaling pathways.^27^ Hub CpG site‐associated genes within significant clinical modules were annotated and analyzed by Kyoto Encyclopedia of Genes and Genomes (KEGG) pathway analysis in Enrichr with default parameters. A *P *< .05 was regarded as significance, and −log_10_ *P* values were plotted and ranked in a bar graph.

### 2.5 Integrated analysis of DCGs and DEGs

The analysis of three microarray datasets was performed using R studio packages, including raw data format transition, missing value interpolation, background calibration, and data quantitative normalization.^28^ After data preprocessing, we defined *P* < .05 and |log 2FC|≥0.25 (FC, fold change) as the threshold for screening differentially methylated‐CpG sites (DCGs). We analyzed differentially expressed genes (DEGs) between the tumor and normal groups using the limma package, with thresholds set at |log2FC|≥1 and *P* ≤ .05. Statistical analysis was performed using the ggstatsplot package in R studio, and the results were visualized using volcano plots or violin plots. The overlapping hub CpG sites between key modules and DCGs were used to annotate the proximate genes in a Venn diagram. The expression levels of annotated genes were further validated in a training dataset (http://www.ncbi.nlm.nih.gov/geo/query/acc.cgi?acc=GSE30784) and a validation dataset (http://www.ncbi.nlm.nih.gov/geo/query/acc.cgi?acc=GSE31056). To explore the relationship between differential expression levels and methylation patterns, the promoter methylation of selected hub genes was analyzed by MethHC (http://methhc.mbc.nctu.edu.tw/php/index.php)^29^ and plotted in a bar graph.

### 2.6 Survival analysis

To explore the potential prognostic value of hub genes, we used GEPIA database (http://gepia.cancer-pku.cn/)^30^ for HNSCC to perform overall survival analysis for multiple‐hub CpG site‐associated genes using Mantel‐Cox tests, and presented the results as a survival heatmap. Overall survival analysis for each CpG site was performed using the Kaplan‐Meier test in the MethSurv database (://biit.cs.ut.ee/methsurv/).^31^ Log‐rank tests were used to measure statistical significance and Log‐rank *P* < .05 was considered as significance.

### 2.7 Patients and samples

A series of 40 OSCC biopsy specimens was prospectively collected and frozen at −80℃ in the Xiangya Hospital, Central South University, Changsha City, China. All subjects gave their informed consent for inclusion before they participated in the study. The study was conducted in accordance with the Declaration of Helsinki, and the protocol was approved by the Ethics Committee of Xiangya Hospital. The clinical and histological information of 40 OSCC patients were summarized in Table S1.

### 2.8 Bisulfite treatment and QMSP

DNA was extracted from all samples with phenol/chloroform and precipitated with ethanol. Sodium bisulfite conversion of genomic DNA was done as previously paper.^11^ The modified DNA was used as a template for real‐time fluorogenic QMSP. The primers used for the each target gene CpG sites (OSR1 cg06509239 and ZNF677 cg18335068) and for the internal reference gene (ACTB) are listed in Table 1. QMSP assay was prepared including 10 μL of 2×SYBR^®^ Green Realtime PCR Master Mix (Cat # QPK‐201, TOYOBO Life Science), 2 μL of bisulfite‐modified genomic DNA, 2 μL of 100‐pmol primers, and completed with 6 μL of DEPC H_2_O. The PCR cycling conditions were as follows: initiated the PCR with a 10 minutes denaturation step at 95℃, followed by 36 cycles: 95℃‐30 seconds (denaturation); 55℃‐30 seconds (annealing); and 72℃‐30 seconds (elongation) on a LightCycler480 (Roche). To determine the relative levels of methylated‐CpG sites in each sample, the values of each CpG sites were normalized against the values of the internal reference gene ACTB to obtain a ratio. The following formula of 2^−ΔΔ^ *^C^* ^t^ method was used: Methylation ratio = 2^−ΔΔ^ *^C^* ^t^/(2^−ΔΔ^ *^C^* ^t^ + 1). ΔΔ*C*t = (Ct _Methyprimer_ − Ct _ACTB_) − (Ct _UnMethyprimer_ − Ct _ACTB_). We also set negative control (use ddH_2_O instead of ACTB primer).

**Table 1:** Primer sequences for QMSP and real‐time PCR

Sequence name	Sequence (5′‐3′)
OSR1‐cg06509239 MethyPrimer	GGGAGAGGTTGGGTTTAAGC
OSR1‐cg06509239 UnMethyPrimer	GGGAGAGGTTGGGTTTAAGT
OSR1‐cg06509239 Reverse primer	ACTAACCCAAACAACAAACT
ZNF677‐cg18335068 MethyPrimer	GAGAGGGTTTAGAGATTTGC
ZNF677‐cg18335068 UnMethyPrimer	GAGAGGGTTTAGAGATTTGT
ZNF677‐cg18335068 Reverse primer	TTACCCTCTTCAAAACAAT
ACTB UnMethyPrimer	GGTGATGGAGGAGGTTTAGTAAG
ACTB UnMethy ReversePrimer	ACCAATAAAACCTACTCCTCCCTTA
OSR1 RT‐PCR forward primer	CGGTGCCTATCCACCCTTC
OSR1 RT‐PCR reverse primer	GCAACGCGCTGAAACCATA
ZNF677 RT‐PCR forward primer	ACAAGCAAGGGATTATCACCAAA
ZNF677 RT‐PCR reverse primer	CAGGCTGTCAAACTTAGGCAT
ACTB RT‐PCR forward primer	CATGTACGTTGCTATCCAGGC
ACTB RT‐PCR reverse primer	CTCCTTAATGTCACGCACGAT
GAPDH RT‐PCR forward primer	GGAGCGAGATCCCTCCAAAAT
GAPDH RT‐PCR reverse primer	GGCTGTTGTCATACTTCTCATGG

Abbreviation: QMSP, quantitative methylation‐specific PCR.

### 2.9 Real‐time PCR

Total RNA was extracted from OSCC tissues by TRIZOL method (Invitrogen) and RNA was reversely transcripted to cDNA by Reverse transcription kit (Thermo Fisher Scientific). Real‐time PCR was performed using 10 μL of SYBR^®^ Green Realtime PCR Master Mix, 2 μL of cDNA, 2 μL of primers, and 6 μL of ddH_2_O on a LightCycler480 to detect the expression of *OSR1* and *ZNF677*, with *ACTB* and *GAPDH* as a normalizing control. The primers sequence for *OSR1*, *ZNF677*, *ACTB,* and *GAPDH* was listed in Table 1.

### 2.10 Correlation scatter plot of selected hub gene expression and CpG methylation patterns

To analyze whether *OSR1* and *ZNF677* expression levels were correlated with methylation of hub CpG sites, a http://www.baidu.com/link?url=JhH_GEQNs1JT-nvMEPZrZVhVOySQohEpKmVDNM1kUcj3Ww88D9uFyW0C5FJSBdo9aI-KDoAc7H5gFu0y6leInNPiAjRhs8SuFwsi7PIW21-Oy3CROOAqaq8kyCb31Vh_%26wd=%26eqid=ae8f119d00008557000000055d9d8524 scatter plot was constructed using the expression and methylation data from MethHC and our OSCC samples. The association was determined by calculating Pearson's correlation coefficient (*R* ^2^). A *P* < .05 was considered to indicate a statistically significant correlation.

## 3 RESULTS

### 3.1 Construction of comethylation network associated with OSCC status by WGCNA

**Figure 1:**
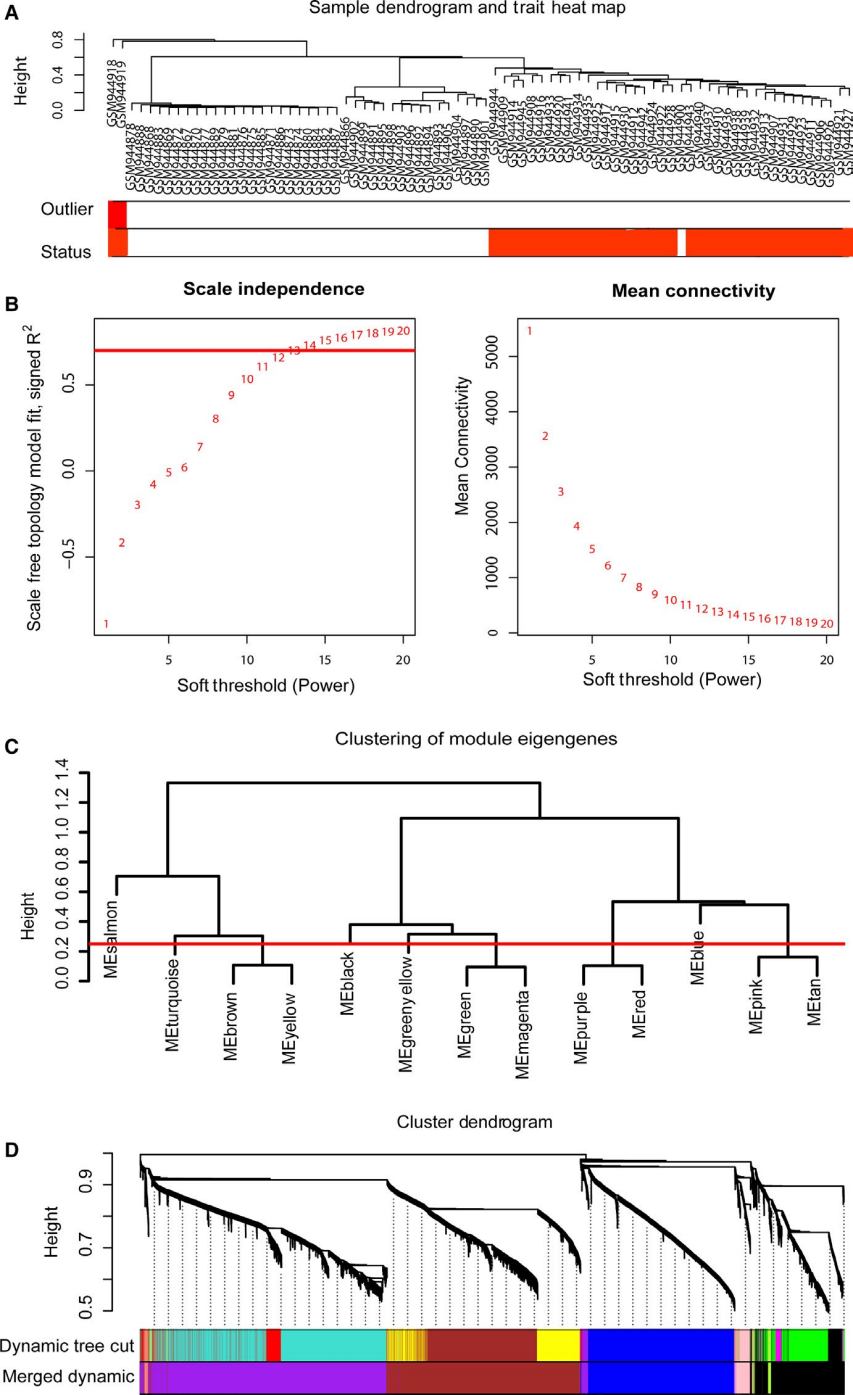
Construction of comethylation network associated with OSCC status by WGCNA. (A) Clustering dendrogram of 80 samples. (B) Analysis of the scale‐free fit index and mean connectivity for various soft‐thresholding powers. Red line represents the appropriate scale‐free topology fit index at 0.75. (C) Dendrogram of consensus module eigengenes obtained by WGCNA on the consensus correlation. The red line at 0.25 indicates the merging threshold; groups of eigengenes below the threshold represent modules whose expression profiles were merged owing to their similarity. (D) Merged modules were identified by the Dynamic Tree Cutting method. Each module was assigned a color as an identifier. Seven modules were generated after merging according to the correlation of modules. OSCC, oral squamous cell carcinoma

A total of 27 578 probes for CpG sites were annotated and matched with OSCC status. In the sample dendrogram and trait heat map, the samples and related clinical information were connected by WGCNA, with two outlier samples (GSM944918 and GSM944919) excluded using the flashClust function (Figure 1A). A “soft‐thresholding procedure” was performed by WGCNA, and a best‐fit cutoff value (β = 13) was selected at the lowest value of the mean connective and the appropriate scale‐free topology fit index (0.75) (Figure 1B). To construct the comethylation network and identify the key module, a hierarchical clustering tree for the module was produced using the best‐fit β‐value of 13 (Figure 1C). The cut line was set at 0.25 to merge similar modules, resulting in 7 merged modules (Figure 1D).

**Figure 2:**
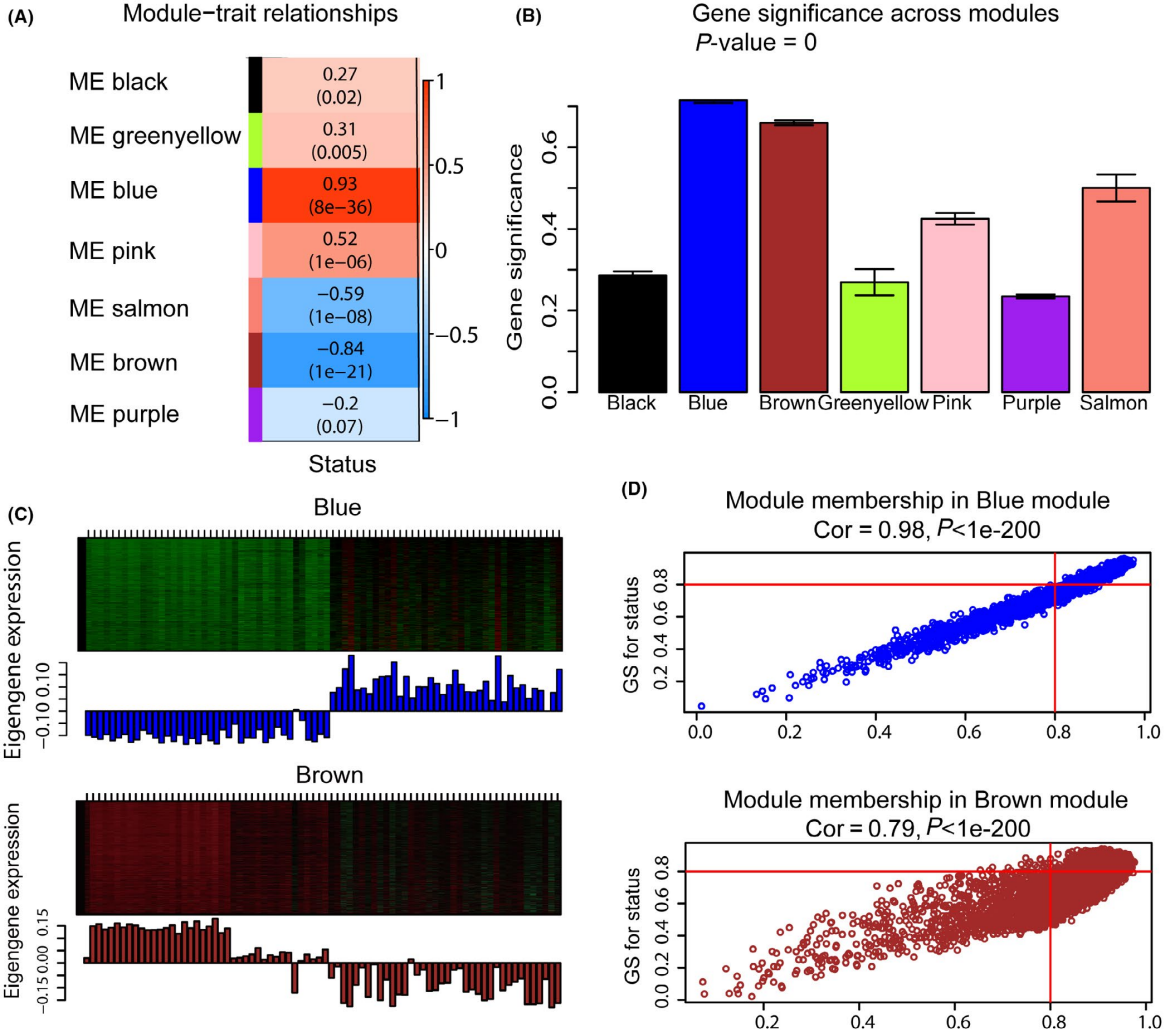
Identification of hub modules associated with OSCC status. (A) Heatmap of the correlation between module eigengenes and OSCC status. Vertical axis represents modules, horizontal axis represents status. (B) Distribution of average GS in modules associated with OSCC status. (C) Heat maps and boxplots showing methylation status of CpG sites within the 2 hub modules. Hypermethylation is represented by red and hypomethylation by green in the heat maps. In the boxplot, the vertical axes represent the methylation ratio and the horizontal axes represent samples. (D) Scatter plot of GS vs MM in the brown module and blue module. Red cutoff lines for MM and GS at 0.8 indicate the criteria used to screen for hub CpG sites. GS, gene significance; MM, module membership; OSCC, oral squamous cell carcinoma

The module‐trait heat map revealed the relationship of OSCC status and methylated‐CpG sites within the modules (Figure 2A,B). The blue module, comprising positively related CpGs, was significantly correlated with OSCC status (*R* = .93 and *P* = 8e‐36). Meanwhile, the brown module was negatively associated with OSCC status (*R*=−.84 and *P* = 1e‐21). The modules with the highest MS were considered to be key modules with respect to OSCC status. The blue and brown modules shown in Figure 2B represent the 2 modules with the highest MS, consistent with the Pearson's correlation index results in the module‐trait heat map.

### 3.2 Screen of hub methylated‐CpG sites within blue and brown modules

The methylation status of CpG sites within the blue and brown modules related to OSCC status is displayed in the heat map and boxplot in Figure 2C. A higher methylation ratio of CpG sites was observed in the blue module for OSCC samples compared with normal samples, with the opposite tendency for the brown module. These results imply that the methylation status of CpG sites within key modules is highly related to clinical characteristics, consistent with the MS and Pearson's correlation index results shown in the module‐trait heat map. To further identify the hub CpG sites within key modules, screening criteria of MM >0.8 and GS >0.8 were used to identify 559 CpG sites from the blue module and 298 CpG sites from the brown module (Table S2).

### 3.3 Screen of hub methylated‐CpG sites and associated genes

**Figure 3:**
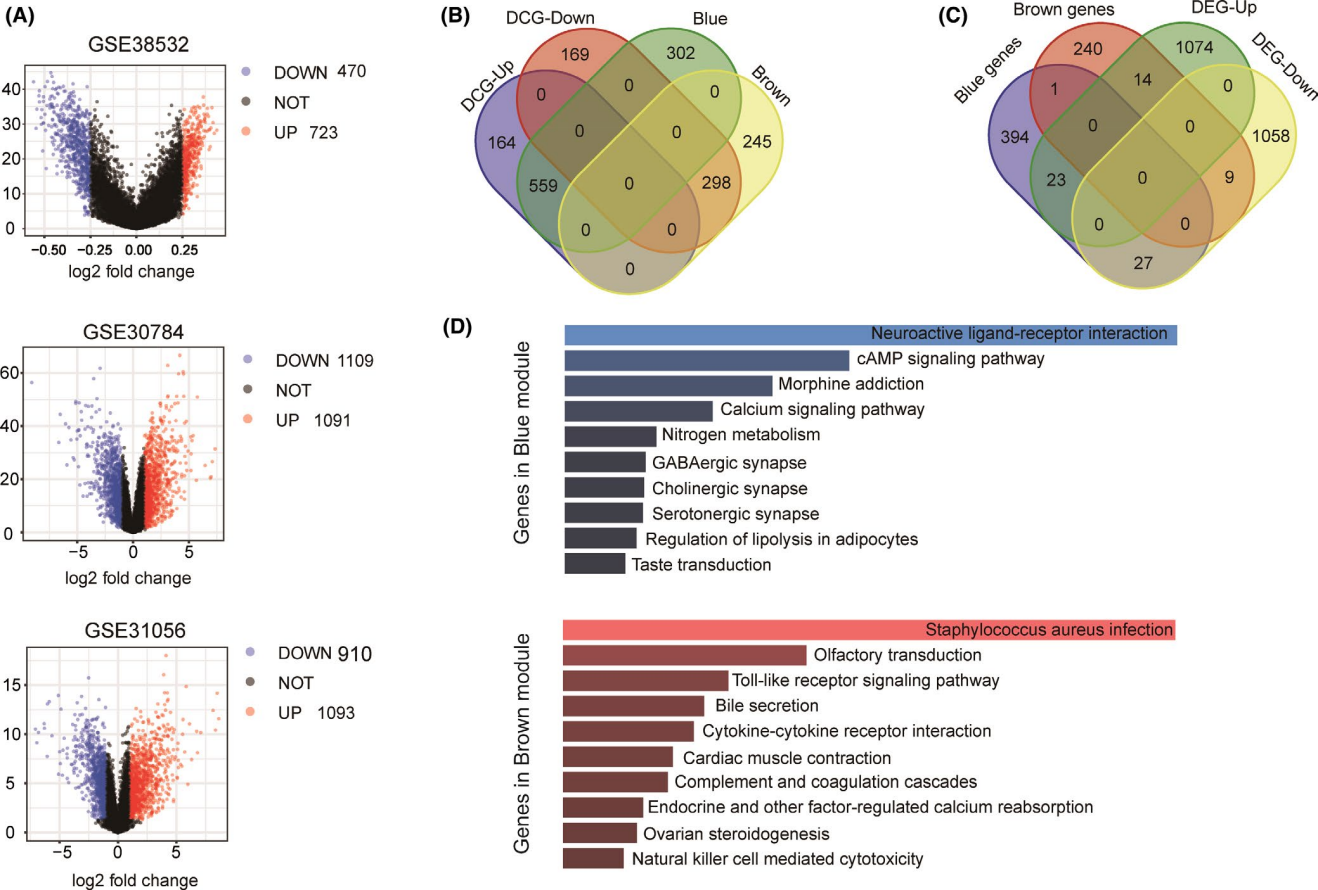
Selection of hub methylated‐CpG sites and associated genes. (A) Volcano plot of DCGs and DEGs. Red dots represent upregulated genes, blue dots represent downregulated genes. (B) Venn diagrams of DCGs and genes in blue and brown modules. (C) Venn diagram of DEGs and DCG‐associated genes in blue and brown modules. (D) Functional analysis of DCG‐associated genes in blue and brown modules showed in boxplot. Top 10 enriched KEGG pathways are ranked by −log10 (*P*). DCGs, differentially methylated‐CpG sites; DEGs, differentially expressed genes; KEGG, Kyoto Encyclopedia of Genes and Genomes

**Figure 4:**
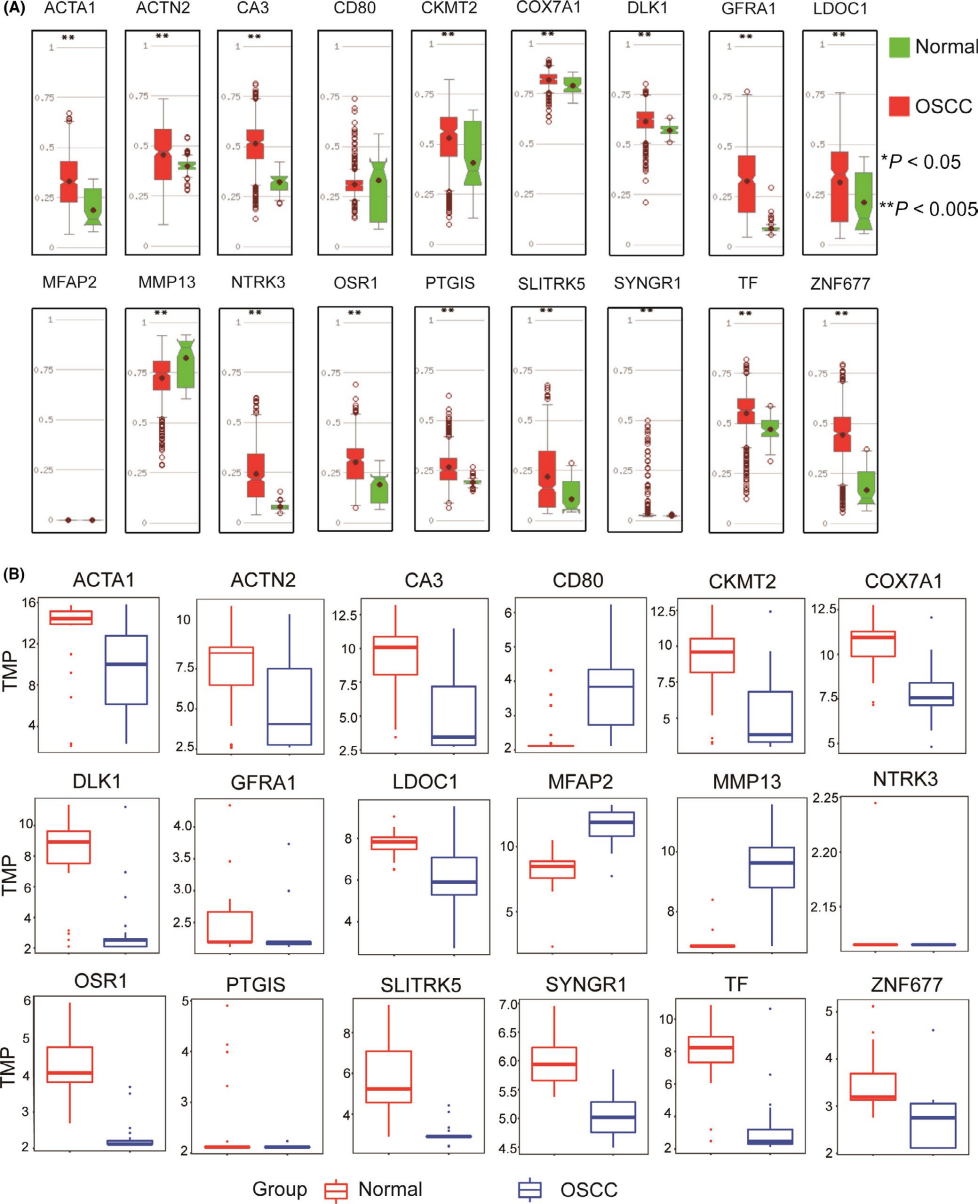
Correlation analysis for selected hub genes between promoter methylation status and expression levels. (A) Boxplots showing promoter methylation status of 18 hub genes, analyzed via MethHC. (B) Boxplots showing expression levels of 18 hub genes from http://www.ncbi.nlm.nih.gov/geo/query/acc.cgi?acc=GSE31056. Normal samples (n = 45) are shown as red box and dots and OSCC samples (n = 167) as blue box and dots. OSCC, oral squamous cell carcinoma

To further screen hub methylated‐CpG sites and associated genes, 3 microarrays (http://www.ncbi.nlm.nih.gov/geo/query/acc.cgi?acc=GSE30784, http://www.ncbi.nlm.nih.gov/geo/query/acc.cgi?acc=GSE31056, and http://www.ncbi.nlm.nih.gov/geo/query/acc.cgi?acc=GSE38532) were used to analyze the DEGs and DCGs. As shown in Figure 3A, A total of 723 hyper‐DCGs and 470 hypo‐DCGs were screened out in the methylation profile of http://www.ncbi.nlm.nih.gov/geo/query/acc.cgi?acc=GSE38532. These DCGs intersected with hub CpG sites within the blue or brown module, resulting in a total of 559 hyper‐DCGs within the blue module and 298 hypo‐DCGs within the brown module, as illustrated in the Venn diagram in Figure 3B. In addition, 559 genes associated with hyper‐DCGs were annotated within the blue module, and 298 genes were found near hypo‐DCGs within the brown module (Table S2), according to the corresponding annotation information. As shown in the volcano plot in Figure 3A, 1091 upregulated DEGs and 1109 downregulated DEGs were found in the http://www.ncbi.nlm.nih.gov/geo/query/acc.cgi?acc=GSE30784 microarray. Figure 3C shows the four groups of genes that overlapped, from which hub genes associated with methylated‐CpG sites were selected. We selected the hub DEGs from the module eigengenes for which expression levels were negatively associated with the methylated ratio of CpG sites. Therefore, among the 4 groups, 27 downregulated DEGs from the blue module and 14 upregulated DEGs from the brown module were identified. The http://www.ncbi.nlm.nih.gov/geo/query/acc.cgi?acc=GSE31056 expression profile was used to validate the differential expression of 41 hub genes in the OSCC samples. Only 18 hub genes had statistically different expression levels in the validation dataset of http://www.ncbi.nlm.nih.gov/geo/query/acc.cgi?acc=GSE31056 in Figure 4B (*P* < .05).

### 3.4 Functional enrichment analysis of hub CpG site‐associated eigengenes from key modules

We performed KEGG enrichment analysis to reveal the functions of the hub genes associated with hyper‐DCGs and hypo‐DCGs in the blue and brown modules. As shown in Figure 3D, hub genes in the blue and brown module were mainly enriched in top 10 signaling pathways, respectively. Some critical pathways of blue module were enriched, such as cAMP signaling pathway and calcium signaling pathway, which are related to cancers.

### 3.5 Differential expression and methylation patterns of hub methylated‐CpG site‐associated genes

To investigate the correlation of differential expression levels and methylation patterns, the promoter methylation of hub methylated‐CpG sites‐associated genes were analyzed using MethHC. Promoter methylation ratios of 15 hub genes were significantly higher in OSCC samples than in normal tissues (*P* < .005) (Figure 4A), and expression levels of 15 hub genes were significantly lower (*P* < .05) (Figure 4B). Only levels of *MMP13* were elevated and had a lower promoter methylation ratio. The expression levels of *MFAP2* and *CD80* were not correlated with their promoter methylation and the 2 genes were excluded in the following analysis. Thus, expression levels of 16 hub genes were negatively correlated with their methylation status of promoter. It implies that promoter methylation of hub methylated‐CpG site‐associated genes affects their expression.

### 3.6 Survival analysis for hub methylated‐CpG sites and their associated genes

**Figure 5:**
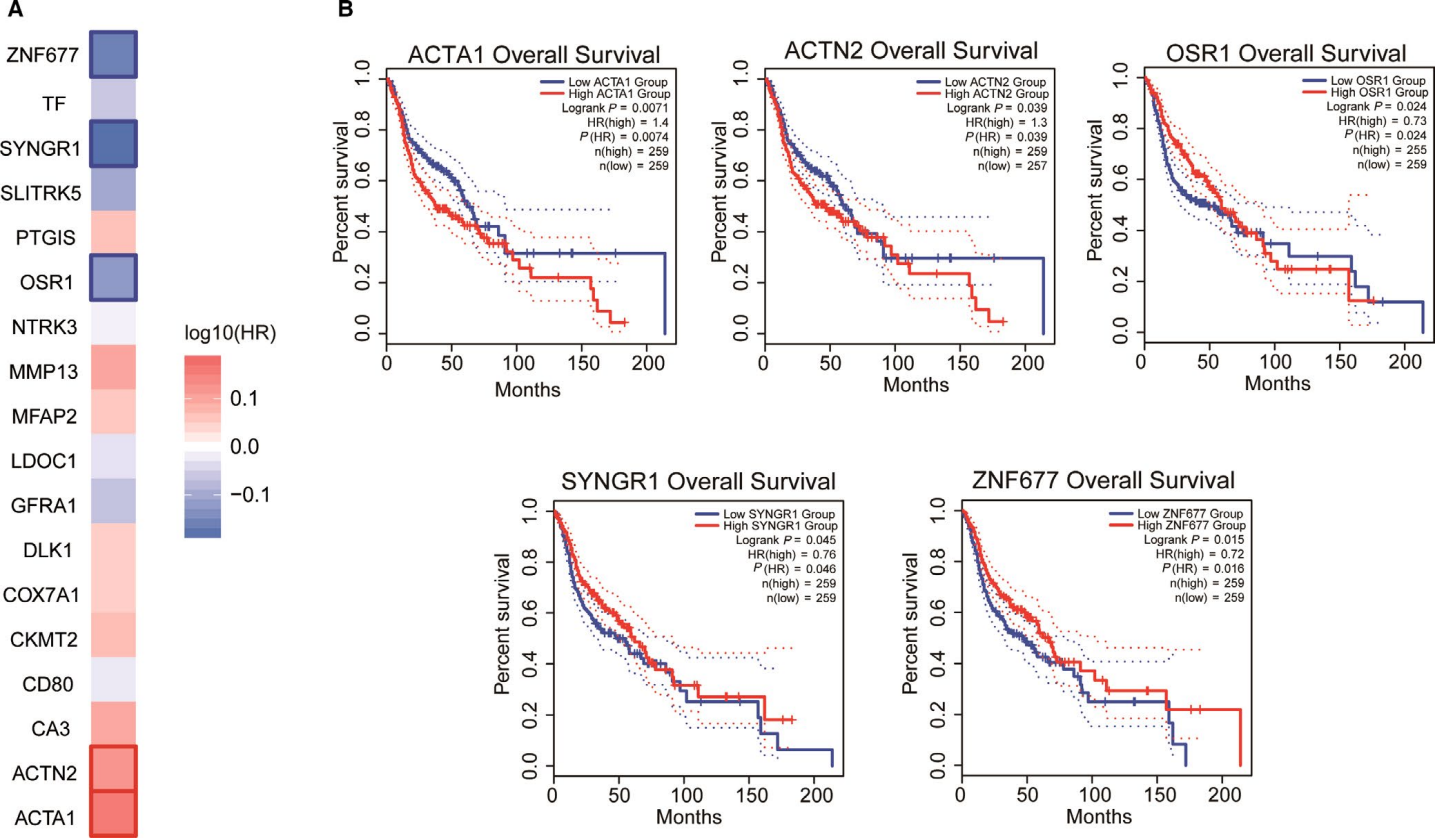
Overall survival analysis of hub genes. (A) Survival heat map for 18 selected hub genes. (B) Kaplan‐Meier curve analysis for *ACTA1*, *ACTN2*, *OSR1*, *SYNGR1*, and *ZNF677* with respect to overall survival in OSCC patients. A log‐rank *P *> .05 was considered to indicate a significant difference. OSCC, oral squamous cell carcinoma

In order to assess whether these hub genes were prognostic indicators, a survival heat map of multiple genes identified by the Mantel‐Cox test was constructed in GEPIA. Figure 5A shows the survival heat map for 18 hub genes and only 5 genes, *ACTA1*, *ACTN2*, *OSR1*, *SYNGR1*, and *ZNF677* had significant overall survival (log‐rank *P* < .05). The Kaplan‐Meier curve showed that low expression of *ACTA1* and *ACTN2* was associated with improved survival, and 3 genes, *OSR1*, *SYNGR1*, and *ZNF677*, were associated with poor survival (Figure 5B).

**Figure 6:**
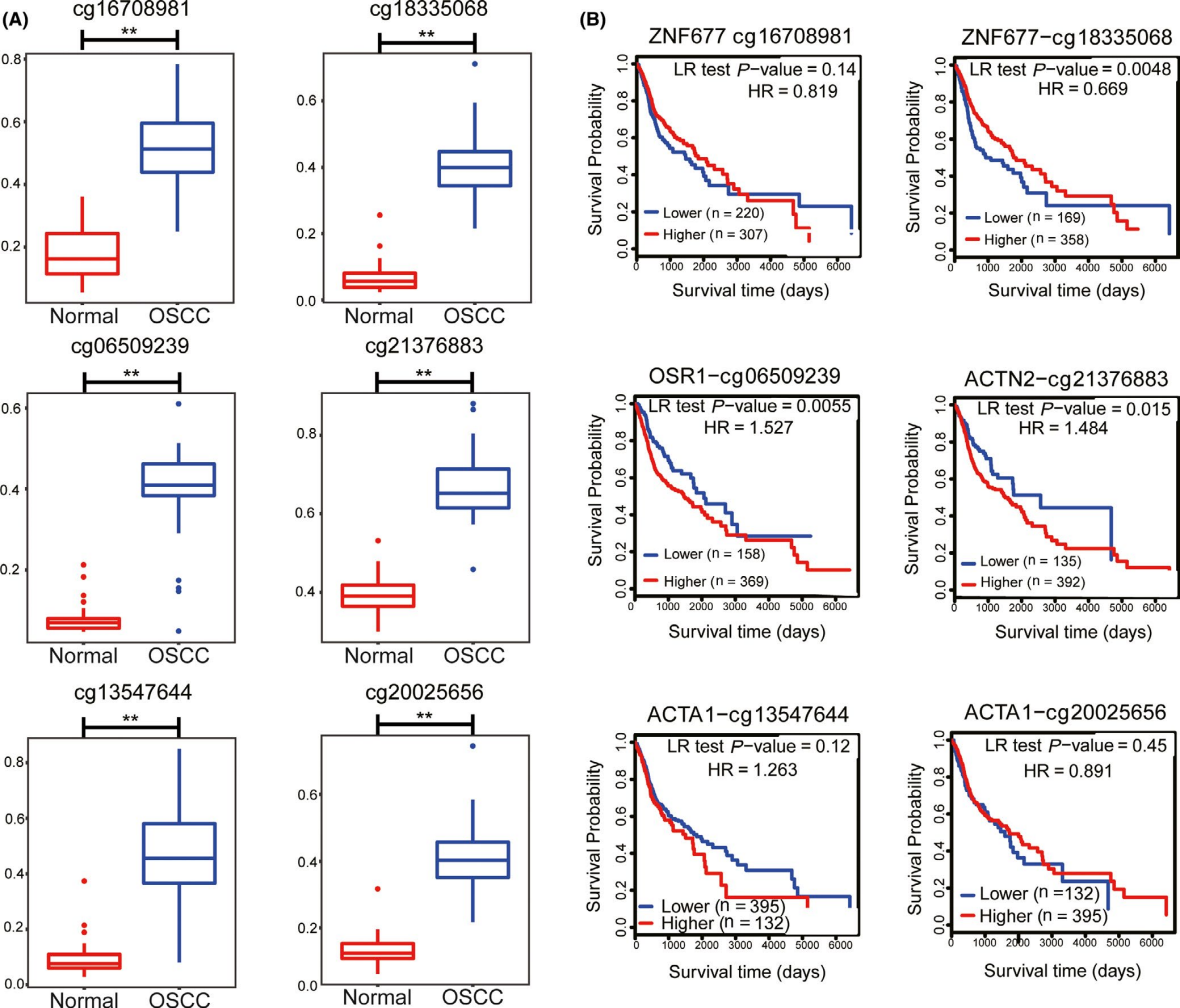
Methylation status of hub CpG sites and overall survival analysis. (A) Hypermethylation patterns of the 6 hub CpG sites in OSCC samples compared with normal samples using data of http://www.ncbi.nlm.nih.gov/geo/query/acc.cgi?acc=GSE38532. Normal samples (n = 40) are shown as red box and OSCC samples (n = 40) as blue box. Y axis represents methylation level. ** (P<0.01) indicates a significant difference. (B) Overall survival analysis of hub CpG sites by Kaplan‐Meier test using MethSurv. A log‐rank *P* < .05 was considered to indicate a significant difference. OSCC, oral squamous cell carcinoma

Seven hub CpG sites from key modules were found to be located near these 5 genes. Hypermethylation patterns of 6 hub CpG sites were found in OSCC samples compared with normal samples (Figure 6A). Kaplan‐Meier curve analysis was performed to obtain the prognostic signature of hub methylated‐CpG sites by log‐rank test. SYNGR1‐cg19713460 was excluded for lack of survival data. Hypermethylation CpG sites ACTN2‐cg21376883 (log‐rank *P* = .015) and OSR1‐cg06509239 (log‐rank *P* = .0055) were associated with poor survival (Figure 6B), whereas hypermethylation CpG site ZNF677‐cg18335068 (log‐rank *P* = .0048) was associated with an improved prognosis. Methylation levels of ZNF677‐cg16708981, ACTA1‐cg13547644, and ACTA1‐cg20025656 were not associated with survival (*P* > .05).

### 3.7 Correlation scatter plot of selected hub genes’ expression and CpG methylation patterns

**Figure 7:**
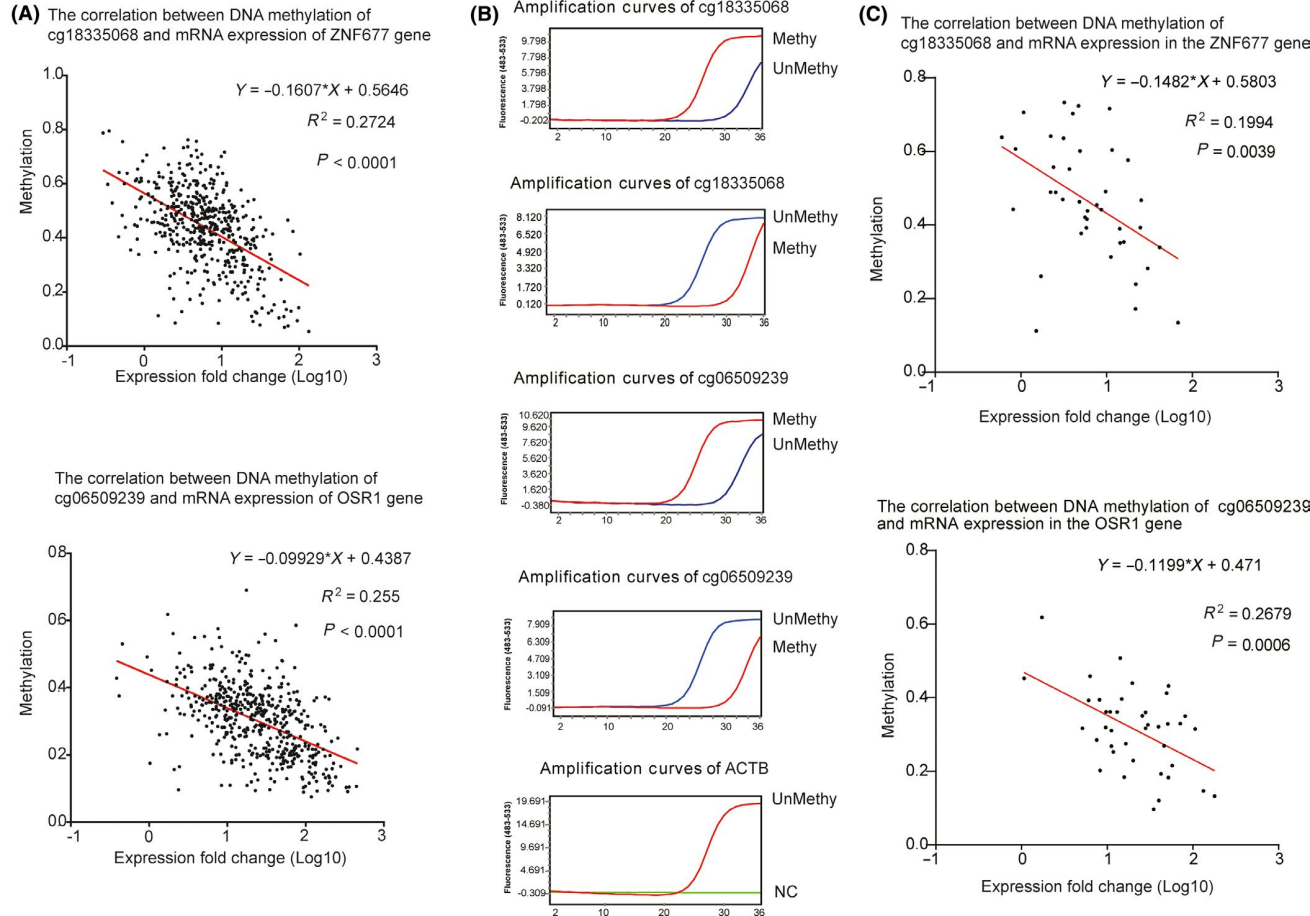
Linear correlation analysis of selected hub CpG sites and mRNA expression levels in MethHC and clinical samples by Pearson's correlation analysis. (A) Linear correlation scatterplot of hub CpG sites in MethHC. *X*‐axis represents mRNA expression fold change (Log10). *Y*‐axis represents methylation ratio. (B) QMSP for the identification of methylation status of CpG sites using 40 OSCC samples from the hospital. Methy represents MehthyPrimer and UnMethy represents UnMethyPrimer. NC means negative control (using ddH_2_O instead of *ACTB* primers). (C) Linear correlation scatterplot of hub CpG sites using 40 OSCC samples. Real‐time PCR was used to determine mRNA levels of *ZNF677* and *OSR1* in 40 OSCC samples from the hospital. *X*‐axis represents mRNA expression fold change (Log10). *Y*‐axis represents methylation ratio. *P* < .05 is statistical significance. Red line indicates the tendency of scatterplot. *R* ^2^ is correlation coefficient. Equation is linear regression equation. OSCC, oral squamous cell carcinoma; QMSP, quantitative methylation‐specific PCR

Linear Pearson's correlation analysis was performed to clarify whether the methylation status of hub CpG sites was correlated with the associated hub genes. In Figure 7A, we used the public data from MethHC and constructed a linear regression scatter plot. The results showed that methylation levels of cg06509239 and cg18335068 were negatively related to *OSR1* (*R* ^2^ = .255) and *ZNF677* (*R* ^2^ = .2724) mRNA levels, respectively. Moreover, we also constructed QMSP assays to detect methylation status of hub CpG sites (Figure 7B) using clinical samples of OSCC, which were further used to build a linear regression scatter plot. In Figure 7C, methylation ratio of cg06509239 (*R* ^2^ = .2679) and cg18335068 (*R* ^2^ = .1994) was also decreased, accompanied by the increase in expression levels of *OSR1* and *ZNF677* (*P* < .05) in a negatively dependent manner. Thus, these findings demonstrate that the decrease in *OSR1* and *ZNF677* expression levels in OSCC samples could be associated with the hypermethylated‐CpG sites.

## 4 DISCUSSION

This study used integrated multiomics analysis to show that methylation patterns of hub CpG sites in OSCC were associated with expression levels of the corresponding genes. To describe the relationships between methylation profiles and OSCC status, we used a modified WGCNA method, weighted gene comethylation network analysis, to screen hub CpG sites from clinical trait‐related modules. In previous studies, many reporters used integrated multiomics analysis to screen hub genes and associated methylated differential genes.^32^, ^33^ The prognostic model based on 5 aberrant differential methylation genes (*PAX9*, *STK33*, *GPR150*, *INSM1*, and *EPHX3*) can be used as independent prognostic biomarkers for predicting the prognosis of patients with head and neck cancer.^32^ Using gene expression profiles of OSCC (http://www.ncbi.nlm.nih.gov/geo/query/acc.cgi?acc=GSE30784 and http://www.ncbi.nlm.nih.gov/geo/query/acc.cgi?acc=GSE38532), 28 upregulated hypomethylated genes and 24 downregulated hypermethylated genes were identified, which were mainly enriched in the biological process of regulation in immune response, PI3K‐AKT and EMT pathways.^33^ WGCNA was used to directly screen out 5 methylation‐associated genes, *CENPV*, *SYTL2*, *OCLN*, *CASD1*, and *TUB*, which may be predictors of survival in patients with OSCC.^34^ Another report characterized a 4‐gene methylation signature consisting of *ZNF10, TMPRSS12*, *ERGIC2*, *and RNF215*, which are associated with efficacy of radiotherapy in HNSCC.^26^

Compared with previous study, a novel aspect of our study was the use of WGCNA to investigate the methylation profile of CpGs in OSCC with a large sample size. This method greatly reduced the multiple testing problems; instead of attempting to relate 27 578 CpGs to OSCC status, we only needed to consider 7 merged comethylation modules. We directly screened out 559 hypermethylation CpG sites and 298 hypomethylation CpG sites from the OSCC status‐associated key module using our screening criteria. The hub methylated‐CpG sites were mapped to the genome and annotated with the nearest genes.

To validate these DCG‐associated genes, two independent gene expression profiles of OSCC were further analyzed. Seven hub genes were statistically differentially expressed in the validating dataset. Hypermethylation of CpG spots in promoters is widely considered to interrupt genomic binding of activating transcription factors or other proteins, and shows a high level of association with gene repression.^35^ Expression levels of 16 of the 18 hub genes (except *CD80* and *MFAP2*) were negatively correlated with their promoter's methylation status. In order to investigate the effect of hub genes on prognosis, a survival heat map was constructed. This showed that 5 hub genes had significant association with survival outcomes, suggesting key roles for these genes in the progression of OSCC. Three hub methylated‐CpG sites (cg21376883, cg06509239, and cg18335068) and their associated genes were characterized by divergent methylated patterns, expression FC, and significant association with survival outcomes. Identification of these unique CpG sites and the associated genes may help to develop novel therapeutic targets for clinical diagnosis in OSCC patients.

ACTN2, an actin‐binding protein, functions as a key transducing molecule in the signaling pathway from IGF receptor I activation to cell membrane microspike production, cell‐cell separation, and cell migration.^36^ *ACTN2* has been identified as a hub gene involved in the focal adhesion signaling pathway and represents a potential new target for treatment of chordomas.^37^ The parafibromin tumor suppressor protein interacts with ACTN2 and ACTN3, suggesting that these proteins probably have antitumor roles.^38^ In this study, ACTN2 expression was decreased in OSCC with the elevated methylation ratio of promoter. However, hypermethylated‐cg21376883 in OSCC samples was not correlated with the low levels of *ACTN2* (Data not shown). cg21376883 was an independent favorable prognostic indicator for OSCC survival (Logrank *P* = .015).

Zinc finger protein 677 (*ZNF677*), located at the chromosomal region 19q13, is involved in transcriptional regulation. *ZNF677* has been shown to have all 3 types of genomic aberration (gain, loss, and copy neutral loss of heterozygosity) in gliomas.^39^ In non–small cell lung cancer (NSCLC), *ZNF677* functions as a tumor suppressor regulating many genes, mainly those involved in growth hormone regulation and interferon signaling.^40^ Moreover, hypermethylation of *ZNF677* probably causes a decrease in *ZNF677* expression, which could serve as a prognostic marker in NSCLC patients.^40^ *ZNF677* also exerts its tumor suppressor functions in thyroid cancer cells through transcriptional repression of *CDKN3* and *HSPB1*, thereby interfering with phosphorylation and activation of Akt via distinct mechanisms.^41^ In this study, we further demonstrated that low expression of *ZNF677* may be associated with poor outcomes in patients (log‐rank *P* = .015); thus, the indicator could be used to monitor cancer progression. Methylation level of ZNF677‐cg18335068 was significantly correlated with its expression using MethHC and our cohort. However, hypermethylation CpG site ZNF677‐cg18335068 (log‐rank *P* = .0048) was associated with an improved prognosis using the data from MethSurv. Because of 2 public data onto overall survival be used, it seems like that hypermethylation of cg18335068 and lower expression of *ZNF677* in OSCC are inconsistent with the tumor suppressor functions in other cancers. Further exploration of the role of *ZNF677* in OSCC and the effect of cg18335068 on the expression of *ZNF677* are still needed to be performed.

The odd‐skipped related 1 (*OSR1*) gene, located on human chromosome 2p24.1, is a transcription factor that plays a part in the regulation of embryonic heart and urogenital development.^42^ *OSR1* methylation is closely related to the development of several different cancer types.^43^ Methylation of *OSR1* has been shown to be a sensitive indicator of the detection of recurrent urothelial cell carcinoma.^44^ In addition, *OSR1* hypermethylation was identified as an independent predictor of poor survival in gastric cancer patients.^45^ *OSR1* also showed clinical potential as a biomarker in lung adenocarcinoma,^46^ owing to the inactivity of the Wnt signaling pathway resulting from a decrease in SOX9 and β‐catenin levels.^47^ Moreover, OSR1 blocks cell migration and invasion through inhibiting the NF‐κB pathway in tongue squamous cell carcinoma.^43^ OSR1 could serve as a novel epigenetic silenced tumor suppressor downregulating invasion and proliferation in renal cell carcinoma.^48^ In this study, *OSR1* methylated cg06509239 was highly correlated with OSCC status and negatively associated with expression levels in MethHC and our cohort. In OSCC, the low levels of *OSR1* were probably downregulated by the high ratio of methylated cg06509239. Thus, *OSR1* expression and its CpG spot cg06509239 could serve as prognostic indicators for OSCC.

In conclusion, the classification schema using multiomics analysis developed in this work represents a screening framework that could be used to identify hub CpG sites and associated genes to serve as diagnostic and prognostic indicators in OSCC patients.

## CONFLICTS OF INTEREST

The authors declare no conflict of interest.

## AUTHOR CONTRIBUTIONS

Qiang Qu was involved in project administration and supervision. Yuxin Dai was in charge of original draft preparation and software. Qiaoli Lv was involved in data curation, investigation, and original draft preparation. Jian Qu was in charge of picture visualization. Hongli Ni and Yongkang Liao were involved in formal analysis. Peng Liu was in charge of data validation.

## Supporting information

Fig S1

Table S1

Table S2

## Data Availability

The data that support the findings of this study are openly available.
