# Supplementary material for: Identification of hub methylated‐CpG sites and associated genes in oral squamous cell carcinoma
Source: Cancer Med. 2020 Mar 10;9(9):3174–87. doi: 10.1002/cam4.2969 (PMC7196066; doi:10.1002/cam4.2969)
Supplement: Supplementary file 1 — Fig S1 [file CAM4-9-3174-s001.docx]

Supplementary Figure. Over-all flowchart about analysis pipeline

Methylation microarray data

Construction WGCNA model

Screen of clinical key modules

Screen of hub methylated-CpG sites and associated gene from key modules

Functional enrichment

analysis of genes

Association analysis of hub genes and methylation of CpG promoters and CpG sites

Overall survival analysis of genes and associated CpG sites

QMSP and RT-PCR detect methylation status of selected CpG sites and associated genes in our samples

Expression microarray data
